# Retrospective evaluation of radiological and clinical outcomes after surgical treatment of proximal femur fractures utilizing TFNA

**DOI:** 10.1007/s00402-022-04704-x

**Published:** 2022-12-02

**Authors:** Friedemann Schneider, Fabian Geir, Christian Koidl, Luise Gehrer, Armin Runer, Rohit Arora

**Affiliations:** 1 University Hospital for Orthopaedics and Traumatology, Medical University of Innsbruck, Innsbruck, Austria; 2 Medical University of Innsbruck, Innsbruck, Austria; 3 Department of Sports Orthopaedics, Technical University of Munich, Munich, Germany

**Keywords:** Femur, Hip fracture, Intramedullary nail, Cement augmentation, TFNA

## Abstract

**Introduction:**

The aim of this study was to evaluate the clinical and radiological outcomes of patients treated with the TFN-Advanced™ Proximal Femoral Nailing system (TFNA¸ DePuy Synthes, West Chester, PA) including intra- and postoperative complications.

**Materials and methods:**

All patients with an acute proximal femur fracture consequently treated with a TFNA between September 2014 and December 2018 were evaluated. Clinical and radiological data were assessed for intra- and postoperative complications, including treatment failure. In addition, intra- and postoperative X-rays were used to determine the position of the implant, and any migration, via tip-apex-distance (TAD) and the caput-collum-diaphyseal angle (CCD). The accuracy of the fracture reduction was rated by both observers according to Baumgartners criteria.

**Results:**

275 consecutive patients (mean age 77.5 ± 14.1; 70.2% female) were included. The predominant OTA/AO fracture classification was 31A2 (140 cases, 50.7%). The average surgical time was 69 min (± 39.8). The reduction quality was good in 253 cases (92.0%) and acceptable in 22 cases (8.0%). In 18 cases, a pre-defined primary outcome parameter (6.5%) was recorded after a mean of 8.2 ± 8.0 months. During the observational period, 19 patients (6.9%) required a total of 23 additional surgeries. Implant removal was not considered a failure in the absence of pain. Significant group differences were observed with younger age (*p* = 0.001), lower Charlson Comorbidity Index (CCI)-score (*p* = 0.041) and lower rate of osteoporosis (*p* = 0.015) in the failure group. There were no cases of cut-out or cut-through among the patients who underwent augmentation as part of osteosynthesis.

**Conclusions:**

Proximal femur fractures treated with the TFNA show low complication rates and high levels of radiological healing. Implant-related complications might be more common in patients with younger age, a lower CCI-score and lower frequency of osteoporosis. Usage of cement augmentation could potentially be beneficial to reduce postoperative cut-through and cut-out.

## Introduction

Proximal femur fractures are frequent injuries, especially in an increasingly aging population. They result in high patient mortality as well as high financial costs for the health system. A further increase in the number of hip fractures and the associated costs is also expected in the future [1, 2].

Trochanteric fractures account for around 50% of all hip fractures in the elderly. Corresponding to the femoral neck fractures, the trochanteric fractures also have a bimodal distribution between high-energy and low-energy trauma, whereby the majority are also caused by low-energy mechanisms [1].

Non-surgical procedures result in prolonged immobility, which in turn is increasingly associated with pneumonia, urinary tract infections, pressure ulcers and deep vein thrombosis. The majority of trochanteric fractures are treated surgically [1].

A biomechanically stable osteosynthesis, which enables immediate mobilization with weight bearing as tolerated, should be achieved to significantly reduce the incidence of postoperative complications [1, 3, 4].

Various types of complications have been described in the literature. Implant-related complications include peri-implant fractures, loss of fixation, fracture non-union, surgical site infection and pain. Nail breakage is a rare condition, commonly associated with pseudarthrosis and may also result in substantial morbidity for the patient [3–7].

Compared to the previous intramedullary nail models, the Trochanteric Fixation Nail—Advanced (TFNA¸ DePuy Synthes, West Chester, PA) was substantially improved with regards to the risk of loss of reduction, iatrogenic fractures, nail breakage and ease of use.

The reduced diameter of the proximal TFNA nail and the lateral flat design of the proximal nail end were specifically designed to reduce pressure to the lateral wall and the head-neck fragment to prevent the risk of loss of reduction. This risk is further reduced by the use of the newly designed Hollow Reamer, which allows for opening of the femur intramedullary canal without radial force generation and therefore reduces the risk of fragment displacement and loss of reduction [8].

Recent clinical studies suggest that if the final position of the distal nail tip is in the anterior half of the medullary canal, this results in anterior encroachment and increased risk for iatrogenic fractures in some patients. The reduction of the radius of curvature (ROC) from 1500–2000 mm to 1000 mm in the TFNA nails (235 mm and longer) addresses this problem [9–11].

Furthermore, although previously addressed, the construct strength and anchorage in osteoporotic bone in the elderly remains a clinical challenge. The TFNA nail is thus made of a Titanium alloy with increased fatigue strength, which allows reducing the nail diameter while still increasing the fatigue endurance load [8]. In addition, three instead of two locking options increase fixation in osteoporotic bone. For the operating room personnel, for the surgeon and for the sterilization department, easy instrument handling is the key for a safe surgical flow and for efficient reprocessing. The TFNA instrumentation has been improved to address the needs of all these stakeholders. In addition, the modularity of the system allows customizing the TFNA system according to the hospital’s, the surgeon’s and the patient's needs. Two fixation options (blade or screw) are available to adapt the treatment to the patient (e.g., screw option in younger patients versus blade option in osteoporotic bone). Additionally, augmentation with Polymethylmethacrylate (PMMA) bone cement is possible for both blade and screw options in cases where additional fixation is desired. In conclusion the new TFNA nail aims at minimizing the occurrence of loss of reduction, improving surgical flow and reducing the duration of surgery by reducing the number of surgical steps [8].

The aim of this study was to evaluate the clinical and radiological outcomes of patients treated with the TFNA and to evaluate intra- and postoperative complications.

## Material and methods

This study was planned as a retrospective data evaluation conducted at a Level 1 trauma center. All patients with an acute proximal femur fracture-diagnosis and consequently treated with a TFNA between September 2014 and December 2018 were extracted from the electronic hospital system (electronic patient chart, surgery program, X-ray software). Inclusion and exclusion criteria are presented in Table 1. The study was conducted according to the guidelines of the Declaration of Helsinki.

**Table 1:** Inclusion and exclusion criteria

Inclusion criteria	Exclusion criteria
Age: ≥ 18—100 years All patients with acute traumatic fractures treated using Trochanteric Fixation Nail Advanced System (TFNA)—with or without PMMA bone cement augmentation AO Classification: pertrochanteric femoral fracture (31-A1 and 31-A2), trochanteric fracture (31-A3), fracture of the trochanteric area (31-A1/A2/A3) with diaphyseal extension or combined fracture of the trochanteric area and the femoral shaft (32-A/B/C) Intraoperative and immediate postoperative X-rays	Age: < 18 years Treatment using other than TFNA implant Prior surgery to the injured hip Non-traumatic fractures (E.g., metastatic fractures) Missing surgery protocol

The primary objective of this study was to evaluate the intra- and postoperative implant-related complication rate. This was assessed by evaluating the patient chart documentation as well as the intra- and postoperative X-rays. No additional X-rays or examinations were performed.

Eight categories of implant-related complications were established, and every case was assigned to one of the following: (1) implant cut-out, (2) implant cut-through, (3) lateral protrusion of the screw or blade with pain, (4) medial migration of the shaft, (5) protrusion including distal anterior cortex, (6) implant breakage, (7) surgical site infection (SSI), (8) other implant-related complications requiring revision surgery.

Furthermore, a rate of additional surgery was calculated.

Mortality was determined by querying the public Tyrolean state register.

The radiological assessment was carried out by two independent, experienced surgeons from the Department of Orthopedics and Trauma Surgery. The values used are the combined mean values. In the case of major measurement differences, a third, experienced surgeon was consulted to reach a consensus.

The immediate postoperative X-rays in anteriorposterior and lateral view were used to determine the position of the blade/screw in the femoral head according to the work by Cleveland et al. [12], which results in nine possible positions (center–center, center–anterior, center–posterior, inferior–anterior, inferior–center, inferior–posterior, superior–anterior, superior–center, superior–posterior).

The TAD was assessed as introduced by Baumgaertner et al. [13]. In short, using an anteroposterior and a lateral radiograph, the TAD is defined as the sum of the distances between the tip of the lag screw to the apex of the femoral head measured in both views [13]. The CCD angle was determined at the intersection of a straight line through the center of the femoral head and the femoral neck and a second straight line through two centrally located points on the femoral shaft, in accordance with Boese et al. [14]. The position of the nail was, according to Schmutz et al. [10], divided into five different positions in lateral X-ray images (far anterior, anterior, centre, posterior, far posterior).

To determine the extent of lateral migration of the blade/screw—head element, the length of the blade/screw protruding laterally from the intramedullary nail was measured in all conducted follow-up X-ray images in anteriorposterior and lateral view. If the shaft diverged medially against the head-neck-fragment, the horizontal distance between the outer edge of the cortex of the femoral shaft and the fracture fragment was measured in the a.p. radiographs. Similarly, for lateralization of the head-neck-fragment a divergence of the cortex was assessed in the a.p. X-ray images by measuring the horizontal distance between the two cortical outer edges.

Fracture reduction was rated according to Baumgartners criteria as “good,” “acceptable” or “poor” with normal or slight valgus alignment on the a.p. X-ray images, less than 20°angulation on the lateral view X-ray images, and less than 4 mm displacement for any fragment. If no agreement was achieved, a third experienced surgeon was asked to find a decision.

“Cut-out” was defined as the cranial penetration of the TFNA blade or screw through the femoral head into the joint, which was usually associated with a pronounced loss of reduction (varus collapse) of the head-neck fragment [16, 17].

**Fig. 1:**
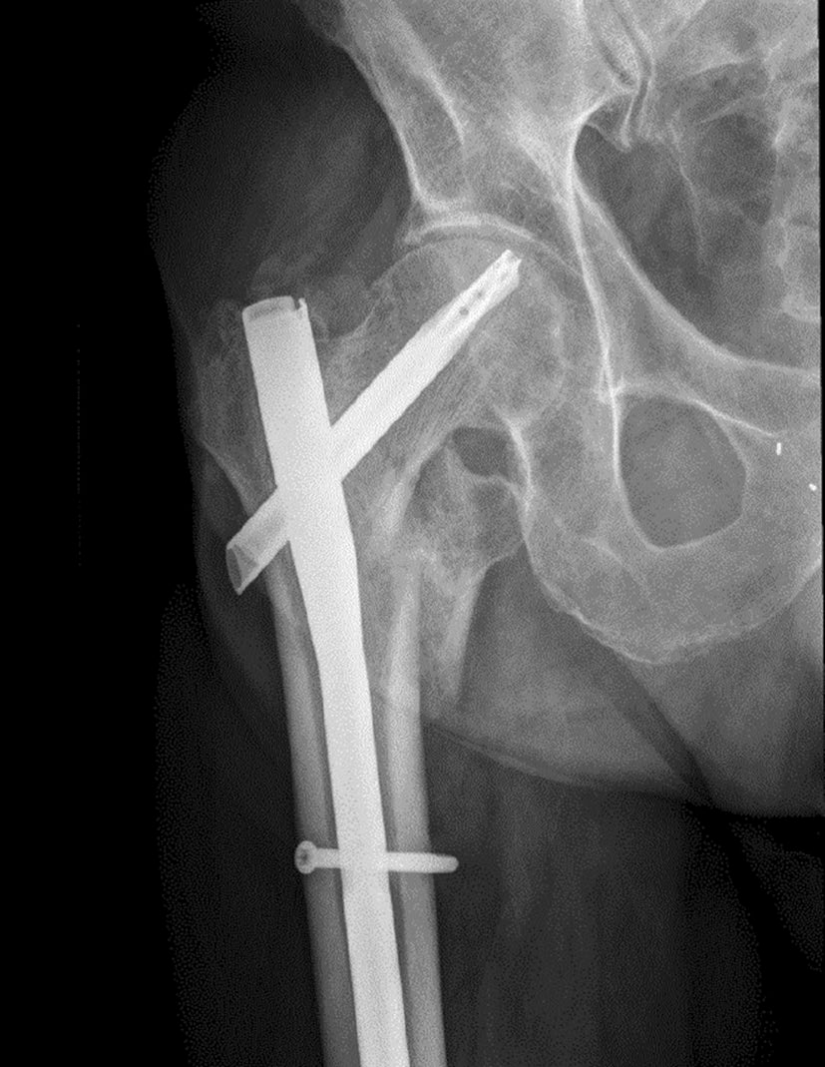
X-ray of a patient with an imminent “Cut-through"-situation

“Cut-through" describes the central penetration of the blade or screw into the joint and occasionally even into the small pelvis without varus collapse. An X-ray of an imminent “cut-through"-situation, occurring in a patient of this dataset, is shown in Fig. 1.

“Lateral protrusion” was defined as the lateral migration of the spiral blade/screw-element commonly resulting in pain or discomfort due to constriction of the soft tissue.

The medial displacement of the femoral shaft in relation to the fracture was defined as "lateral migration of the head-neck-fragment.

All data were analyzed using SPSS v.26 (IBM Corp.). For continuous and normal distributed data, the Student’s *T *test was applied to determine differences between both groups. For ordinal or non-normally distributed data, the Mann–Whitney *U *test was used. Related pre- and postoperative data were compared using the Wilcoxon signed-rank test. A Pearson Chi-square test was performed to compare dichotomous variables. Significance level was set to 0.05 (two- sided) with 95% confidence intervals (CI).

## Results

Within the observation period a total of 275 consecutive patients were treated with intramedullary fixation of an acute proximal femur fracture-diagnosis with a TFNA. The mean follow-up was 6.5 months. The predominant OTA/AO fracture classification was 31A2, as described in Table 2. The incision above the tip of the trochanter was the most frequently chosen approach, as recommended by the manufacturer DePuy Synthes [8].

The reduction quality was good in 253 cases (92.0%) and acceptable in 22 cases (8.0%). In 34 patients (13.9%) a so-called “far anterior” position of the distal nail tip was found. This was not associated with a higher complication rate.

202 patients (73.2%) were treated with a short TFNA (170 mm, 200 mm and 235 mm), the remaining 74 patients (26.8%) with a long TFNA (260–480 mm). A blade was used in 264 patients (96.0%), a screw in 11 cases (4.0%). The most frequently used blade length was 95 mm. Similarly, the most used screws had a length of 95 mm or 100 mm. The position of the blade/screw-element in the femoral head was found to be in a center–center position in 188 cases (68.4%). Additional augmentation with PMMA cement (Traumacem V+, DePuy Synthes, West Chester, PA) was considered in patients with poor bone quality. In total cement augmentation was used in 68 cases (25.0%) (Table 2).

**Table 2:** Patient characteristics and group compositions

	All patients	No complication	With complication	*p* value
Total number (*n*)	275	257	18	
Mean age (SD)	77.5 (14.1)	78.3 (13.5)	65.6 (17.4)	0.001
Sex				
Female	193 (70.2%)	183 (71.2%)	10 (55.6%)	
Male	82 (29.8%)	74 (28.8%)	8 (44.4%)	
Fracture classification				
31A1	85 (30.9%)	80 (31.1%)	5 (27.8%)	
31A2	140 (50.9%)	133 (51.8%)	7 (38.9%)	
31A3	43 (15.6%)	38 (14.8%)	5 (27.8%)	
32A	1 (0.4%)	1 (0.4%)	0 (0.0%)	
Combination injuries	6 (2.2%)	5 (1.9%)	1 (5.6%)	
CCI-score (median)	5.8	6	4.5	0.041
Osteoporosis	139 (50.4%)	135 (52.5%)	4 (22.2%)	0.015
Mean duration of surgery in min. (SD)	69.3 (39.8)	103.0 (58.8)	67.4(37.5)	0.007
Augmentation	68 (25.0%)	67 (26.1%)	1 (5.6%)	0.052
Reduction quality acc. to Baumgartner				
Good	253 (92%)	237 (92.2%)	16 (88.8%)	
Acceptable	22 (8%)	20 (8%)	2 (11.2%)	
Intraop. TAD in mm	21.0 (± 6.1)	21.0 (+ -6.1)	20.4 (+ -6.3)	
Follow-up interval^a^				
4–6 weeks	151 (54.7%)			
2–3 months	196 (71%)			
4–6 months	134 (48.6%)			
1 year	46 (16.7%)			

^a^Number and percentage of patients who had a follow-up at each interval

A total of 18 complications (6.5%) were recorded. With a total of 5 cases the most common complication reported were lateral protrusions of the blade or screw followed by pseudoarthrosis. Signs of bone healing could be observed at 3 months postoperatively in 98.0% (*n* = 192). One patient with a pending cut-out about two months after surgery declined surgical intervention and reached boney healing at about 5 months. Revision surgery had to be performed in three patients with pseudarthrosis after 8 and 9 months, respectively. All primary outcome parameter are listed in Table 3, all additional surgeries in Table 4. During the observational period 19 patients (6.9%) required a total of 23 additional surgeries, of which most were implant removals (two at the request of the patient, six with consequent arthroplasty). One patient showed a loosening of the blade with imminent cut-out during follow-up and was offered surgical revision but decided not to have further surgery. Finally, the fracture healed after about 5 months with no further loosening of the implant.

**Table 3:** Primary outcome parameter

	All patients (*n* = 275)
Lateral protrusion (screw/blade)	5 (1.8%)
Pseudarthrosis	3 (1.1%)
Surgical site infection (SSI)	2 (0.7%)
Persisting pain	2 (0.7%)
Cut-through	2 (0.7%)
Cut-out	2 (0.7%)
Malrotation	1 (0.4%)
Bolt loosening	1 (0.4%)
Total	18 (6.5%)
Mean time to complication in months	8.2 ± 8.0
Death within 12 months postoperative	34 (12.4%)

**Table 4:** Additional surgery

Implant removals	13
With consequent arthroplasty Due to lateral protrusion and pain	6 2
At patients’ request without pain	2
At patients’ request with pain/discomfort Due to surgical site infection With pseudarthrosis revision and re-osteosynthesis	1 1 1
Replacement of a blade Removal of bony fragments Wound revision Derotation Removal of loosened bolt	4 2 2 1 1
Total	23 surgeries (19 patients, 6.9%)

Significant differences between the groups with and without complication were observed with regard to age (*p* = 0.001), CCI-score (*p* = 0.041) and frequency of an osteoporosis diagnosis (*p* = 0.015). There were no cases of cut-out or cut-through among the patients who underwent augmentation as part of osteosynthesis.

## Discussion

The primary finding of the present study is that with the use of the TFNA, surgeons could achieve good results with regard to reduction quality and implant positioning, as well as low rates of postoperative complications.

Sambandam et al. reported that cephalomedullary nails are the most widely used fracture fixation devices for trochanteric fractures in North America. Similarly, European studies show an increasing trend in use of cephalomedullary nails. Given the variety of designs of the nails and conflicting data on their complication rate, a differentiated consideration is worthwhile [18].

One of the latest intramedullary fixation systems, the TFNA system from DePuySynthes, was investigated in our study. Due to the novelty of the system mentioned, there is scarce available evidence on this implant.

As in many other studies on intramedullary nail fixation for proximal femoral fractures, significantly more women were included in this study [5, 7, 17, 19–21]. Although no significant relationship between sex and complication-rate after surgery with a TFNA-system could be shown, this study confirms a greater general risk for women to end up with a proximal femur fracture.

Eighteen appearences of pre-defined primary outcome parameters were registered in this study, representing a total of 6.5% of all surgeries performed. This fits in well with the existing literature, in which the overall rate of mechanical complications has been described to be up to 20%. [5, 6, 20].

It must be noted that previous studies have reported various definitions of “failure”. Any reason that led to additional surgery was defined as failure e.g., in the publication of Matre et al. [22]. In this study, a distinction was made between different complication scenarios, which were pre-defined as primary outcome parameters, and e.g., mere implant removals without any complaints in young patients, which were not considered a complication in this study.

The rather high one-year mortality rate in this study of 12.4% has to be seen in the context of the general morbidity of the underlying patient population. The calculated CCI-score of 5.8 (SD 2.8) corresponds to an estimated 10-year survival rate of about 2%, due to a significantly higher average age compared to the general population as well as the high number of concomitant diseases.

Nevertheless, in comparison to other cohorts, this study shows a relatively young collective, which is particularly noticeable through some young high-energy trauma patients. The complication group included two patients in their thirties, who significantly reduced the mean age and, with moderate symptoms overall, had to have the implant removed due to irritations of the tractus or due to an interfering bony fragment. In this regard, the age-dependent results should be interpreted with caution, since they relate to a heterogeneous collective.

As less osteoporosis and a lower number of points in the Charlson Comorbidity Index was associated with a higher complication rate, it might be assumed that individuals with fewer illnesses and a more active lifestyle put more stress on the osteosynthesis and the implant, and likely have a lower perioperative risk for further surgical treatments (e.g., total hip replacements) in the case of progredient joint wear. More frequent irritations and loosening of the implant in people with a more active lifestyle and better muscle status also seems quite understandable, especially in view of the nevertheless high rate of e.g., osteoporosis in the appropriate age group.

Similarly, the average younger age of the patients as a risk factor fits well with the result of the low Charlson Comorbidity Index [23, 24], since a younger age is usually associated with fewer illnesses and more activity. This may result in the therapeutic approach of developing an adapted behavioral concept for the patient to further reduce complications. For this purpose, prospective studies would be necessary to determine the exact relation of younger age, better health condition and the risk of intra- and postoperative complications.

The relevance of TAD, which is often described in the literature as a predictive factor for failure, remains unclear in this study, as there was no significant difference in TAD between both groups [13, 17].

No cut-outs or cut-throughs were observed in patients with additional cement augmentation. There was only one postoperative complication when additional augmentation was used. In this case, the patient who had experienced an intertrochanteric femur fracture, and was 81 at the time of the initial injury and osteosynthesis, developed a pseudarthrosis, which resulted in an implant fracture at eight months after primary surgery. The pseudoarthrosis was revised by implant removal and consequent arthroplasty. A recent meta-analysis by Rompen et al. [25] suggested that cement augmentation in fixation of trochanteric femur fractures leads to fewer complications, re-operations and shorter hospital stays. To further evaluate the effect of augmentation, prospective studies with a larger proportion of cement-augmented patients would be urgently needed, since in our study only 25% (68 patients) had received cement augmentation.

Schmutz et al. found that a greater curvature of the TFNA leads to reduced cortical entrapment [10]. However, only TFNA with a length between 320–400 mm were examined in this study. In our study, a so-called “far anterior” position was found in 34 patients (13.93%), but this was not associated with a higher complication rate.

Our study has several limitations. Due to the retrospective study design, the available data were not normally distributed, and a selection bias cannot be fully excluded. However, the patients were included at the largest public hospital in the region which is freely accessible to all patient groups. Therefore, it was possible to reach a large sample size which, to the best of the authors' knowledge, is in this regards the largest in literature to date. Finally, it should be mentioned that only 16.7% of the patients included in the study participated in the last follow-up appointment 1 year after surgery and, in general, elderly people might have been released earlier if an age-appropriate result was achieved with possibly already pre-existing limited mobility. This is a phenomenon that can be commonly perceived in long-term studies, but which clearly reduces the informative value of the late follow-up examinations. It might be assumed that patients who recovered well did not see any need for further controls.

## Conclusions

Proximal femur fractures treated with the TFNA show low complication rates and high levels of radiological healing. Implant-related complications might be more common in patients with younger age, a lower CCI-score and lower frequency of osteoporosis. Usage of cement augmentation could potentially be beneficial to reduce postoperative cut-through and cut-out.

## Data Availability

The data that support the findings of this study are available on request from the corresponding author [FS]. The data are not publicly available due to them containing information that could compromise research participant privacy/consent.
